# Pathogenetic Mechanisms of Liver-Associated Injuries, Management, and Current Challenges in COVID-19 Patients

**DOI:** 10.3390/biom13010099

**Published:** 2023-01-03

**Authors:** Muhammad Naeem, Naheed Bano, Saba Manzoor, Aftab Ahmad, Nayla Munawar, Saiful Izwan Abd Razak, Tze Yan Lee, Sutha Devaraj, Abu Hazafa

**Affiliations:** 1 College of Life Science, Hebei Normal University, Shijiazhuang 050024, China; 2 Department of Fisheries and Aquaculture, Muhammad Nawaz Sharif University of Agriculture, Multan 60000, Pakistan; 3 Department of Zoology, University of Sialkot, Sialkot 51310, Pakistan; 4 Biochemistry/Center for Advanced Studies in Agriculture and Food Security (CAS-AFS), University of Agriculture, Faisalabad 38040, Pakistan; 5 Department of Chemistry, College of Science, United Arab Emirates University, Al-Ain 15551, United Arab Emirates; 6 BioInspired Device and Tissue Engineering Research Group (BioInspira), Department of Biomedical Engineering and Health Sciences, Faculty of Electrical Engineering, Universiti Teknologi Malaysia, Johor Bahru 81310, Malaysia; 7 Sports Innovation & Technology Centre, Institute of Human Centred Engineering, Universiti Teknologi Malaysia, Johor Bahru 81310, Malaysia; 8 School of Liberal Arts, Science and Technology (PUScLST) Perdana University, Suite 9.2, 9th Floor, Wisma Chase Perdana, Changkat Semantan Damansara Heights, Kuala Lumpur 50490, Malaysia; 9 Faculty of Medicine, AIMST University, Bedong 08100, Malaysia; 10 Department of Medicine, Surgery and Dentistry, “Scuola Medica Salernitana”, University of Salerno, 84081 Baronissi, Italy; 11 Department of Biochemistry, University of Agriculture Faisalabad, Faisalabad 38040, Pakistan

**Keywords:** liver injury, SARS-CoV-2, liver transplants, chronic liver disease, COVID-19, NFALD, management, interleukins, liver cirrhosis

## Abstract

The global outbreak of COVID-19 possesses serious challenges and adverse impacts for patients with progression of chronic liver disease and has become a major threat to public health. COVID-19 patients have a high risk of lung injury and multiorgan dysfunction that remains a major challenge to hepatology. COVID-19 patients and those with liver injury exhibit clinical manifestations, including elevation in ALT, AST, GGT, bilirubin, TNF-α, and IL-6 and reduction in the levels of CD4 and CD8. Liver injury in COVID-19 patients is induced through multiple factors, including a direct attack of SARS-CoV-2 on liver hepatocytes, hypoxia reperfusion dysfunction, cytokine release syndrome, drug-induced hepatotoxicity caused by lopinavir and ritonavir, immune-mediated inflammation, renin-angiotensin system, and coagulopathy. Cellular and molecular mechanisms underlying liver dysfunction are not fully understood in severe COVID-19 attacks. High mortality and the development of chronic liver diseases such as cirrhosis, alcoholic liver disease, autoimmune hepatitis, nonalcoholic fatty liver disease, and hepatocellular carcinoma are also associated with patients with liver damage. COVID-19 patients with preexisting or developing liver disease should be managed. They often need hospitalization and medication, especially in conjunction with liver transplants. In the present review, we highlight the attack of SARS-CoV-2 on liver hepatocytes by exploring the cellular and molecular events underlying the pathophysiological mechanisms in COVID-19 patients with liver injury. We also discuss the development of chronic liver diseases during the progression of SARS-CoV-2 replication. Lastly, we explore management principles in COVID-19 patients with liver injury and liver transplantation.

## 1. Introduction

The Coronavirus Disease 2019 (COVID-19) is one of the most contagious and infectious diseases caused by an attack of Severe Acute Respiratory Syndrome Coronavirus-2 (SARS-CoV-2) [1]. According to the latest survey released on 14th August 2022 and conducted by Johns Hopkins University, approximately 589 million people were diagnosed with COVID-19 worldwide due to the COVID-19 pandemic, of which 6.4 million died due to serious complications, including liver injury (https://coronavirus.jhu.edu/map.html; accessed on 18 August 2022). The most common site of SARS-CoV-2 attack is lung tissue. Still, most patients with COVID-19 develop mild or asymptomatic symptoms, and the severe form leads to increased mortality due to multiorgan dysfunction complications, especially liver injury, myocardial dysfunction, acute coronary syndromes, and neurological disorders, in addition to respiratory failure [2,3]. SARS-CoV-2 directly attacks liver hepatocytes resulting in abnormal liver function, but the mechanism of action remains unclear. However, no strong evidence exists to indicate which contributing factors play an important role in liver injury in COVID-19 patients. Interaction between preexisting liver disease and COVID-19 has not been reported yet.

Liver injury is one of the major causes of death in COVID-19 and is characterized by direct damage to liver hepatocytes and results in abnormal liver function. Levels of different liver enzymes are elevated in the case of liver damage, such as alanine aminotransferase (ALT), aspartate aminotransferase (AST), alkaline phosphatase (ALP), and gamma-glutamyl transferase (GGT). Elevated levels of these enzymes indicate severe liver damage that increases the risk of mortality among COVID-19 patients, while the levels of other liver proteins, such as albumin, decrease in COVID-19 patients [4]. However, some studies revealed that high CRP levels are also an important indicator of liver injury in COVID-19 patients [5]. Recent studies revealed that elevated levels of TNF-α and IL-6 were observed in COVID-19 patients with injury [6]. Histopathological studies showed liver necrosis, portal fibrosis, poor infiltration in the ductular lobe, and microvesicular steatosis as a result [3].

SARS-CoV-2 entry into liver hepatocytes occurs through ACE2 receptors; however, the mechanism of action is poorly understood [7]. Some factors contribute to liver injury in COVID-19 patients. This form of liver injury in COVID-19 patients is induced through multiple factors, including a direct attack of SARS-CoV-2 on liver hepatocytes, hypoxia reperfusion dysfunction, cytokine release syndrome, drug-induced liver injury caused by lopinavir and ritonavir, immune-mediated inflammation, and coagulopathy [8]. Excessive use of hepatotoxic medications for COVID-19 increases the risk of drug-induced liver damage [9]. An increase in alcohol consumption and unhealthy food items might increase the severity of liver disease [10]. A recent study revealed that advancing age, diabetes, and obesity increase mortality risk in COVID-19 patients with chronic liver disease [11]. Pathophysiological mechanisms and immunological responses in patients with liver injury suffering from COVID-19 are not fully understood. Understanding the pathophysiological mechanisms underlying liver dysfunction in COVID-19 patients is necessary to design novel therapies.

COVID-19 patients have a high risk of developing liver damage, which remains a major challenge [12]. Recent studies showed that COVID-19 patients have compromised immune systems and have a risk for diseases such as autoimmune hepatitis, alcohol-related liver disease, and non-fatty acid liver disease [13]. Such patients are more susceptible to COVID-19 infection and need intensive care, hospitalization, and vaccination.

In this review, we highlight the entry routes and attacking mode of SARS-CoV-2 on liver hepatocytes by exploring the cellular and molecular events underlying the pathophysiological mechanisms in COVID-19 patients with liver injury. We also discuss the recent challenges faced during the pandemic in developing chronic liver diseases during the progression of SARS-CoV-2 replication. Lastly, we explore the management principles for COVID-19 patients with liver injury and liver transplantation.

## 2. Pathophysiological Mechanisms of COVID-19-Associated Liver Injury

**Figure 1:**
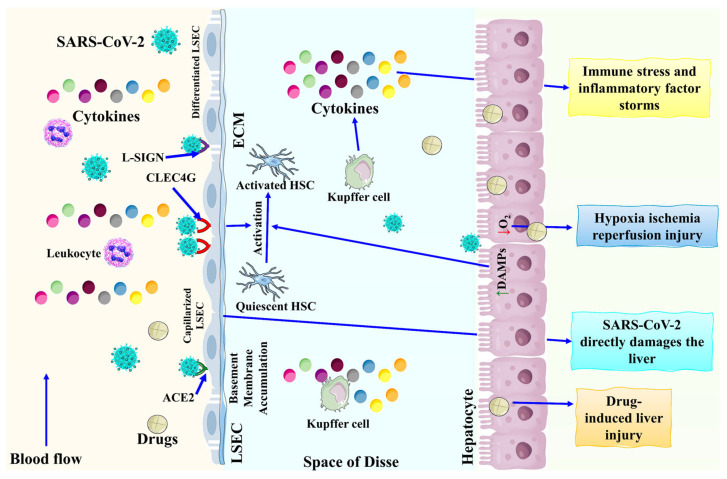
Mechanism of action of SARS-CoV-2 related liver damage. LSEC: Liver sinusoidal endothelial cells; L-SIGN: Liver/lymph node-specific intercellular adhesion molecule-3-grabbing integrin; CLEC4G: C-type lectin domain family 4 member G; DAMP: Danger-associated molecular patterns. This figure is reproduced from Li et al. [14] (Attribution-NonCommercial-NoDerivatives 4.0 International (CC BY-NC-ND 4.0)).

Patients with liver disease are more susceptible to COVID-19 infection and have a high risk of developing metabolic diseases that increase the severity of liver disease. Pathophysiological features in such patients are categorized into different mechanisms followed by direct damage to liver hepatocytes, ischemia/hypoxia hepatic injury, cytokine release syndrome (CRS) and renin-angiotensin-aldosterone system (RAS), and drug-induced liver injury (see Figure 1).

### 2.1. Hepatotropism Mechanisms of Liver Injury

Angiotensin-converting enzyme 2 (ACE2) is a family of receptors expressed on the host cell during replication of SARS-CoV-2 infection, thus providing a gateway for viral entry into the host cell [15]. In vitro studies revealed that trypsin facilitates the binding of ACE2 receptors to the spike protein of SARS-CoV-2 and promotes their replication in a host cell. Trypsin is also expressed in epithelial cells of the liver and facilitates viral entry into the host cell, although the expression of ACE2 is low in liver cells [16]. Single-cell RNA sequencing revealed that the expression of ACE2 receptors is higher in cholangiocytes compared to hepatocytes [17]. Cholangiocytes are epithelial cells found on the surface of the bile duct [18]. Recent studies showed that SARS-CoV-2 proliferates in a culture of liver ductal organoids and binds to cholangiocytes, damaging the liver and causing liver injury due to SARS-CoV-2 viral replication [19]. A severe attack of the COVID-19 virus blocks the bile duct, ultimately leading to bile duct dysfunction, revealing the mechanism of liver injury.

Another study reported that the expression of ACE2 was high in liver fibrosis, indicating a liver injury that directed the hepatic tropism of SARS-CoV-2 [20]. In vitro studies showed that scavenger receptor B type 1 (SR-B1) also facilitates the interaction and binding of ACE-2 with coronavirus [21]. It was shown that the spike protein of SARS-CoV-2 also binds to transmembrane serine protease 2 (TMPRSS2) and pairs with the basic amino acid-cleaving enzyme (FURIN). These receptors showed expression in liver cells, thus facilitating the attack of SARS-CoV-2 on liver cells, leading to liver injury [22]. Zhao et al. [19] demonstrated that the expression of ACE2-expressing and TMPRSS2 in human liver ductal organoids indicates SARS-CoV-2 infection and blockage of epithelium of the bile duct [19].

### 2.2. Hepatic Ischemia and Hypoxia Reperfusion Injury

**Figure 2:**
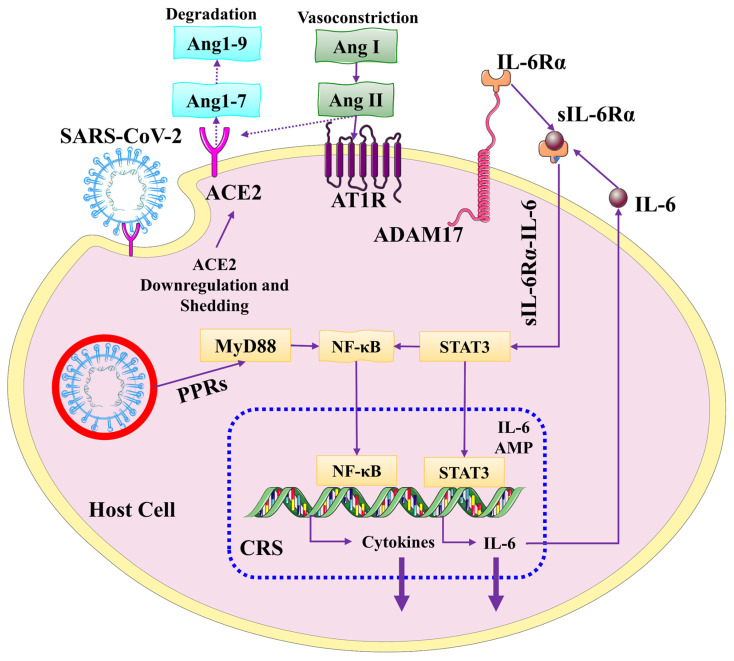
The association between cytokine release syndrome (CRS) and renin-angiotensin system (RAS). ACE2 is a key anti-regulatory enzyme that converts Ang II to Ang1-7. Following viral complex endocytosis, ACE2 is downregulated and lost from the surface of the host cell, resulting in angiotensin II accumulation. Through the AT1R-metalloprotease 17 (ADAM17) axis, Ang II constricts blood vessels and acts as a proinflammatory cytokine. The membrane form of IL-6Ra can be cleaved by ADAM17, resulting in soluble IL-6R that binds to IL-6 and activates STAT3. This trans-signalling causes CRS, which results in the production of several proinflammatory cytokines and chemokines, including increased IL-6. As a result, the feedback loop of the IL-6 amplifier (IL-6 Amp) may act as a switch to launch “cytokine storms.” This figure is reproduced from Li et al. [26] after permission from Springer Nature (license no. 5445930573563).

COVID-19-associated hypoxia also impairs liver functions, which ultimately increases the risk of liver failure [23]. Liver hypoxia is one of the major causes of hepatic damage caused by microvascular thrombosis and lung injury/respiratory failure due to gas exchange defects in the lungs [24]. In the case of a severe COVID-19 attack, an inadequate supply of oxygen to hepatocytes promotes necrosis, which in turn, promotes the development of myocardial infarction. The risk of heart attack and respiratory failure is ultimately increased, and the lung’s function is impaired by decreasing oxygen uptake (see Figure 2) [25].

Hypoxic hepatitis is another major cause of liver injury, also known as ischemic hepatitis [27]. The ischemic injury also contributes to gut damage by promoting intestinal endotoxin. Ischemic injury promotes hepatocyte death caused by disturbances in metabolic processes, resulting in the depletion of ATP, low oxygen supply, more glycogen consumption, and lipid metabolism disorders [28]. Reperfusion injury also contributes to liver injury, followed by metabolic processes and immune reaction disturbances. DAMPs are released as a result of cell death, which activates the complement system and ROS production. These immune reactions lead to the activation of a variety of cells in the liver, including dendritic cells, neutrophils, Kupffer, NK, and T cells [28,29,30].

**Figure 3:**
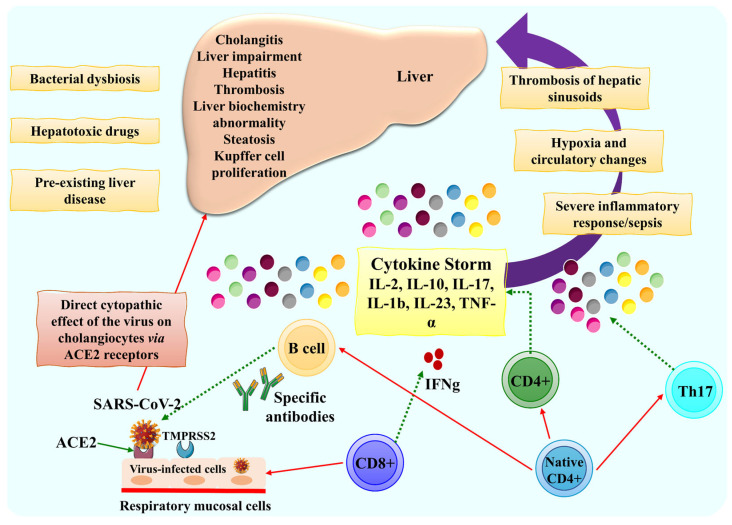
During a COVID-19 infection, liver dysfunction is defined by the detrimental consequences of cytokine storms (severe inflammation, thrombosis, hypoxia, and so on). Cholangitis and cytopathic effects are events that occur during COVID-19 infection as part of acute liver injury. However, acute cholangiocyte injury may be a contributing cause of post-COVID-19 cholestatic liver dysfunction, a topic beyond the scope of the current review. This figure is reproduced from Taneva et al. [31] (Creative Commons Attribution NonCommercial (CC BY-NC 4.0)).

Liver injury is mainly followed by hepatic ischemia/hypoxia and abnormal coagulation mechanisms. A series of inflammatory reactions and activation of immune cells usually follow abnormal coagulation in COVID-19 patients. Monocytes move to the endothelial cells and release tissue factors that activate the extrinsic coagulation pathway. These inflammatory reactions promote fibrin deposition and ultimately endorse blood clotting. Neutrophils move to the sites of infection and release NETs that activate the coagulation pathway by triggering a series of inflammatory reactions. This results in platelet activation to aid blood clotting (see Figure 3) [31].

### 2.3. A Mechanism for Hyper-Inflammation of CRS and RAS Livery Injury

Cytokines are inflammatory biomarkers that play an important role in the severity of COVID-19 patients presenting with complaints of liver injury. Different kinds of inflammatory cytokines are involved in liver injuries, such as IL-6, IL-10, and C-reactive protein (CRP), a well-known inflammatory biomarker. Different studies reported that among inflammatory cytokines, IL-6 is an early indicator in the acute phase in COVID-19 patients, indicating liver injury [32].

Cytokine storm syndrome is one of the most frequently occurring syndromes in COVID-19-infected patients and affects the functions of multiple organs, such as the liver, brain, and lungs. Cytokine release syndrome is caused by the massive release of cytokines that leads to SIRS and ARDS. Increased concentrations of cytokines lead to liver injury, acute respiratory distress syndrome, and brain disorders. A recent study revealed that COVID-19 patients have elevated levels of biomarkers such as CRP, LDH, and IL-6 in case of extreme cytokine storm syndrome [5].

**Figure 4:**
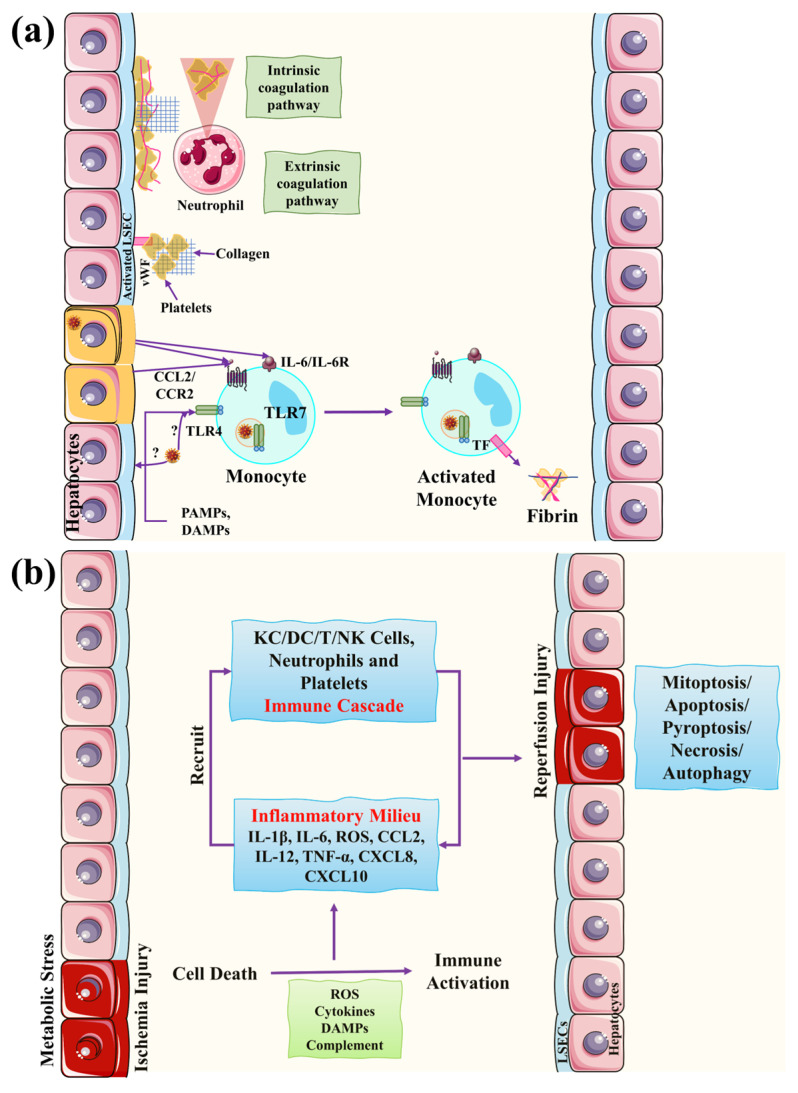
COVID-19-associated liver damage mechanisms of impaired coagulation and hepatic ischemia/hypoxia reperfusion injury. (**a**) In those with severe COVID-19 and hepatic impairment, coagulation problems have been associated with negative results. Monocytes are drawn to endothelial cells in response to proinflammatory stimuli and create tissue factors (TFs), which activate an extrinsic coagulation pathway, resulting in fibrin deposition and blood clotting. Neutrophils are attracted to infection sites early and create neutrophil extracellular traps (NETs), which start a chain reaction of inflammatory responses and activate the contact coagulation pathway, binding and activating platelets to enhance blood clotting. (**b**) Hepatic ischemia/hypoxia reperfusion damage occurs in two stages: ischemia-induced cell injury and reperfusion-induced inflammatory response. Ischemic injury, which is a localized process of cellular metabolic anomalies, initiates hepatocyte cell death. Reperfusion damage, which occurs following ischemia injury, is produced by metabolic anomalies and a strong inflammatory, immunological response that includes both direct and indirect cytotoxic pathways. This figure is reproduced from Li et al. [26] after permission from Springer Nature (license no. 5445930573563).

In the liver, IL-6 is involved in tissue regeneration and involved in metabolic functions, followed by cis- and trans-signaling [33]. In cis-signaling, binding of IL6 with IL-6R results in a stable complex and the formation of a gp130 dimer that regulates the downstream signaling mediated by JAKs and STAT3 pathways that lead to CRS. Expression of mIL-6R is limited to hepatocytes; thus, liver hepatocytes respond to IL-6 signaling [34]. In trans-signaling, binding of IL6 with IL-6R results in a stable complex and the formation of a gp130 dimer that regulates the downstream signaling mediated by JAKs and STAT3 pathways activated in a variety of cells that express mIL-6R and result in the cytokine storm. This type of signaling is mainly involved in a large variety of secretions, including IL-6, chemokines, and proinflammatory cytokines [35]. Thus, hyperinflammation caused by CRS in COVID-19 patients with liver injury can be reversed by targeting IL-6 signaling (see Figure 4).

ACE2 is a group of enzymes involved in the degradation of Ang II to Ang1-7, which is important for the inactivation of RAS. Angiotensin II performs a dual function in liver cells. It acts as a vasoconstrictor and proinflammatory cytokine through the activation of AT1R-metalloprotease 17 (ADAM17), which cleaves the IL-6Rα bound to the surface of the membrane, thus generating the IL-6R that binds to IL-6 and ultimately activates the STAT3 pathway. This mechanism follows trans-signaling and is mainly involved in a large variety of secretions, including IL-6, chemokines, and proinflammatory cytokines. Therefore, IL-6 acts as an inflammatory switch to initiate the signal for cytokine storm [26].

A recent study showed that low levels of CD4^+^T cells, high levels of IL-6, and inflammatory cytokines in COVID-19 patients increase the risk of severe liver injury [36]. Another proteomic-based study involving COVID-19 patients reported high levels of RIG-I, TNF-α, and IL1R in liver tissues, indicating liver injury and expression of these biomarkers mediating the NF-κB pathway [37]. These changes promote the systemic inflammatory response that leads to circulatory dysfunction. On the other hand, a low oxygen supply leads to hypotension, hypoxia, and blood clotting disorders. Histopathological examination of COVID-19 with severe liver injury showed hepatocyte necrosis, thrombosis, mononuclear infiltration, and vascular congestion [38].

### 2.4. Drug-Induced Liver Injury

Drug-induced hepatotoxicity mechanism mainly involves oxidative stress, insulin resistance, mitochondrial dysfunction, and lipid dystrophy [39]. COVID-19 patients also suffer from fever, and cold, so antipyretic drugs containing the acetaminophen ring are used. High-dose concentrations of antipyretic drugs induce liver toxicity and increase the risk of other metabolic diseases. Some antiviral drugs are frequently used alone or in combination, but most of them increase the risk of liver damage [40]. Therefore, there is a need to be cautious about the use of some drugs causing hepatotoxicity.

Excessive use of azithromycin can damage liver hepatocytes and increases the risk of jaundice [41]. Antiviral drugs like lopinavir/ritonavir increase liver injury chances by fourfold compared to other drugs. In some cases, COVID-19 patients received multiple doses of antiviral drugs such as lopinavir/ritonavir, oseltamivir, and abidol. Clinical results of these antiviral drugs showed that they caused liver injury in patients. Antiviral drugs or corticosteroids should be closely monitored in COVID-19 patients with abnormal LFTs [4]. Tocilizumab is also used along with corticosteroids for COVID-19 patients under a ventilator. Tocilizumab is used as an immune suppressive drug, binds to interleukin-6 (IL-6), and blocks its activity. Tocilizumab also activates the replication of hepatotropic viruses and cannot cause direct liver damage. Corticosteroids mainly suppress cytotoxic T cells and activate the replication of HBV, thus induce liver damage (see Table 1) [42,43].

### 2.5. Mitochondrial Dysfunctional Liver Injury

Mitochondrial dysfunction is another cause of liver injury in patients with COVID-19. A study showed that the severity of SARS-CoV-2 infection disrupts mitochondrial activity. In a severe attack of SARS-CoV-2 on liver hepatocytes, oxidative damage to the mitochondrial membrane results in the production of ROS species [49]. Ahmed et al. [50] reported the biological activity of mitochondrial cristae in liver cells diagnosed with COVID-19. They found that abnormalities were observed in mitochondrial cristae that impaired liver function and increased the risk of non-alcoholic fatty liver disease (NFALD) in COVID-19 patients. Another cause of liver injury is the existing liver disease, non-alcoholic steatohepatitis (NASH), in COVID-19 patients with impaired mitochondrial activity. Further study is required to differentiate the mechanisms of NFALD and NASH with impaired mitochondrial activity in severe COVID-19 attacks [50,51].

## 3. Chronic Liver Disease in COVID-19 Patients

COVID-19 infection in patients with chronic liver disease places the patient at greater risk for severe COVID-19 illness. Different factors and pathological features of liver disease in COVID-19 are given below:

### 3.1. Non-Alcoholic Fatty Liver Disease (NAFLD)

Obesity is one of the potential risk factors among COVID-19 patients that causes NAFLD. Inflammatory cytokines such as IL-6, produced in large amounts in such patients, cause severe inflammation and cytokine storm [52]. Different studies showed that obesity prolongs the stay of patients with liver injury diagnosed with COVID-19. Hu et al. [53] investigated a case study involving 58 COVID-19 patients with obesity. These patients gained more fat during hospitalization. Since the expression of ACE2 is enriched in adipocytes and obese people have more ACE2-expressing cells, they are more vulnerable to COVID-19 infection. This activates immunological reactions and results in poor liver function. The authors conclude that obesity is a predisposing factor that increases the risk of other metabolic disorders [53,54]. Another study by Meijnikman et al. [55] revealed that upregulation of ACE2 alleviates the risk of NAFLD in COVID-19 patients as it promotes the development of fat storage in liver and visceral adipose tissues. Storage fat stimulates the penetration of viral particles into liver cells, thus increasing the chance of liver damage [55]. Another retrospective cohort study involving 202 COVID-19 patients with NAFLD had a higher rate of liver disease progression [56]. Another study reported that patients with NAFLD had a higher chance of developing COVID-19 disease [56].

### 3.2. Alcohol-Related Liver Disease (ARLD)

A recent study revealed that ARLD contributes to liver injury in COVID-19 patients [57]. Alcohol consumption is a cause of liver injury, but the extracting mechanism of action of ARLD in COVID-19 patients is poorly understood. However, different studies revealed that poor nutritional status and compromised immune systems increase the chance of ARLD in COVID-19 patients [58]. The Charlson Comorbidity Index indicated that the mortality rate among COVID-19 patients with ARLD in hospitalized patients is significantly different from other localities and non-hospitalized patients. The outcomes depend on the healthcare resources available to manage COVID-19 patients with ARLD. These findings suggested that a high incidence of alcohol drinking might be a potential risk for ARLD in COVID-19 patients. Hospital-based admissions and healthcare allocation for COVID-19 patients with developing ARLD are potential strategies to reduce the risk of liver injury [59,60].

### 3.3. Liver Cirrhosis and Hepatocellular Carcinoma

**Figure 5:**
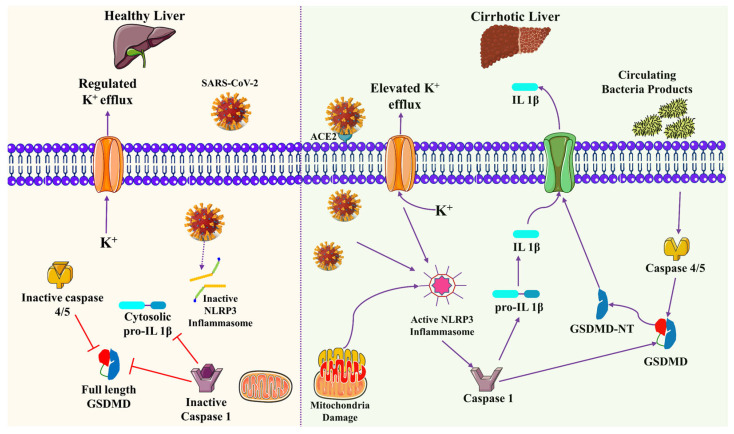
Cirrhosis-related upregulation of hepatocyte inflammasome signaling predisposes increased cell death after SARS-CoV-2 infection. Bacterial substances (such as lipopolysaccharide) bind to and activate caspases-4/5 in cirrhosis (right panel), causing the dimeric protein Gasdermin-D to be cleaved (GSDMD). The N-terminus of GSDMD migrates to the plasma membrane, where it generates holes that allow damage-associated molecular patterns and electrolytes to flow uncontrollably. NLRP3 assembly is also triggered by K^+^ efflux and mitochondrial injury, which activates caspase-1 and results in pro-IL1 processing. Virus proteins attach to the previously synthesized NLRP3, initiating downstream pathways during SARS-Cov-2 infection. In contrast, the absence of the ACE2 receptor in healthy livers (left panel) delays SARS-CoV-2 entry into cells. NLRP3 is present but inactive, preventing proinflammatory caspase activation and processing GSDMD and pro-IL1. This figure is reproduced from Luo et al. [65] (Attribution 4.0 International (CC BY 4.0)).

COVID-19 patients have a high risk of developing cirrhosis as these patients have compromised immune systems and are susceptible to other infections. A recent study revealed that COVID-19 patients with cirrhosis have a higher mortality rate than non-cirrhosis liver disease. The severity of liver cirrhosis is associated with high-risk mortality among COVID-19 patients (see Figure 5) [61]. A case study with 50 patients diagnosed with COVID-19 had a mortality rate of 40%. The study revealed that COVID-19 patients were categorized in Child-Pugh (CP) A, B, and Class C liver cirrhosis with mortality rates of 24%, 43%, and 63%, respectively [57]. COVID-19 patients have a high chance of developing hepatocellular carcinoma (HCC). COVID-19 patients are more susceptible to inflammatory responses as they secrete excessive IL-6 cytokines and have a high chance of developing post-hepatectomy liver failure (PHLF) following hepatectomy [62,63]. Patients with cirrhosis who develop COVID-19 illness have a higher risk of fatal outcomes. These patients need proper management and hospital-based medical treatment [64].

### 3.4. Liver Transplantation

Liver transplant recipients have high risks of liver injury and graft rejection with progressing COVID-19. Some liver transplant patients may have other metabolic complications in addition to liver injury. Such patients have compromised immune systems and high mortality rates. A recent study investigated 1522 patients diagnosed with positive COVID-19 under liver transplantation with a mortality rate of 17.4%. Transplant recipients showed about 2.3% graft dysfunction [66]. Another study reported that 80% of liver transplant recipients with positive COVID-19 needed intensive care and were admitted to the hospital, while 20% of patients needed a ventilator for survival, and the mortality rate was about 17% [67]. Such patients should be monitored with proper medical resources to crossmatch donor and recipient transplants. It will be helpful for the detection of graft rejection at an early stage [68]. Such patients should be vaccinated, and post-exposure prophylaxis should be implemented in high-risk individuals. COVID-19 patients with chronic liver disease shortage were selected for liver transplant surgery, as these patients have a risk of acute liver failure. These patients have higher viral load and high infectivity rates than healthy and non-infected individuals. Liver transplant patients with a compromised immune system have a high risk of mortality when diagnosed with COVID-19 [69].

## 4. Management of Liver Injury in COVID-19 Patients

COVID-19 patients with chronic liver injury need appropriate management and medication. Such patients exhibit symptoms of liver damage and are hospitalized to quantify the viral load, verify liver function, and study other inflammatory markers. Antiviral therapy should be continued in patients with autoimmune liver diseases. Liver transplant patients need intensive care. Appropriate medication should be given to such patients in addition to COVID-19 treatment. The patients should be monitored regularly, and treatment should be carried out in the presence of an expert physician and healthcare staff. Moreover, proper attention should be given to recently diagnosed COVID-19 patients with developing liver disease, as these patients have a risk of liver damage and other complications [70,71].

### 4.1. Non-Alcoholic Fatty Liver Disease (NAFLD)

NAFLD patients with severe COVID-19 infection may have a high risk of developing metabolic diseases such as obesity and diabetes. Such patients should be hospitalized, and monitoring liver biomarkers, glucose assessment, blood pressure, and cholesterol levels should be done carefully to reduce the severity of NAFLD–COVID-19 [72]. Proper medications should be continued in such patients in order to reduce adiponectin or block the secretion of lipid mediators that cause severe inflammation in obese patients. NAFLD patients with COVID-19 infection need additional medical therapy [73,74]. ANGPTL3 is a potential regulator of lipid metabolism. Monoclonal antibodies are widely used for targeting fatty acid compounds in obese individuals. One of the newly discovered antibodies, VHH-Fc, is effective in treating NAFLD, which binds to ANGPTL3 (Angiopoietin-like protein-3) and inhibits its activity [75]. Some corticoids and methotrexate are used for the transition of fatty liver and are thus effective for the treatment of NAFLD [39]. Although monoclonal antibody treatment is effective for NAFLD, some potential drugs are currently under clinical trials and will be available in clinical practice after successful approval.

### 4.2. Autoimmune Hepatitis

Antiviral therapy should be recommended for patients co-infected with HBV/HCV before using an antiviral drug combination for COVID-19 infection [76]. Patients infected with hepatitis B and C also take medical therapy to inhibit viral replication to maintain a healthy lifestyle. Nucleoside analogs should be continued for the inactivation of the hepatitis B virus, as their discontinuation may result in the reactivation of HBV [77]. Some antiviral drugs for COVID-19 have direct interactions with HCV drugs, such as protease inhibitors that showed drug–drug interactions with lopinavir/ritonavir. Thus, drug combinations in HCV must be monitored regularly and prescribed by a physician [76]. Regular serological testing of hepatitis B and C for all COVID-19 patients should be carried out in the presence of an expert analyst, as false positive results can increase the spread of viral infection. COVID-19 patients diagnosed with hepatitis B and C should be hospitalized in separate isolation rooms, and proper medical attention should be given in the presence of an expert physician and healthcare staff [78]. Patients infected with hepatitis A also have a risk of developing liver disease. Such patients should take regular medication and hygienic precautions and avoid large gatherings during the pandemic period [79].

### 4.3. Liver Cirrhosis and Hepatocellular Carcinoma (HCC)

Patients with cirrhosis also have a chance of developing HCC. Such patients need proper medication and regulation of HCC through ultrasound testing and alpha-fetoprotein (AFP) after every six months. It will be helpful for the physician to access patient history and medication to reduce the risk of HCC [80]. A delay in HCC detection leads to the development of severe liver injury. Radiotherapy, immunotherapy, and medical attention should be needed for patients newly diagnosed with HCC [81]. COVID-19 patients with a chronic stage of HCC should be treated with tyrosine kinase inhibitors that may continue with the advice of a physician [82,83]. Priority is given to patients diagnosed at an early stage with HCC. Such patients should be allocated to separate rooms, and medical resources should be provided regularly. Monitoring and surveillance of patients should be carried out as guided by health regulatory authorities or WHO [83]. Proper medical attention should be given to older patients diagnosed with COVID-19. Such patients have a high chance of developing liver injury and risk liver damage due to the poor function of hepatocytes. Moreover, the functions of liver biomarkers for all COVID-19 patients should be monitored carefully with an advanced automated system, as antiviral drugs given to target viral replication have several toxic effects on the liver and can cause liver damage if not monitored regularly. Therefore, anti-viral drugs should be started after accessing the results of liver biomarkers and viral load [84,85].

## 5. Current Challenges of Liver Injury in COVID-19 Patients

The global burden of chronic liver diseases such as autoimmune hepatitis, cirrhosis, non-alcoholic fatty liver disease, and alcohol-related liver disease is high and affects a major part of the human population. However, COVID-19 disease progression status in preexisting liver disease has not yet been studied and remains a challenge for clinical practice [86]. Most liver disease patients, including excessive alcohol users and those with viral infections (HBV/HCV), are at high risk of COVID-19, but the therapeutic efficacy of many drugs is still limited. Obesity is one of the major causes of death in COVID-19 with NAFLD as it leads to the development of metabolic diseases that prolong the period of hospitalization of such patients, which is also a major challenge in clinical therapy [13].

During the pandemic of COVID-19, cases of liver injury patients have increased, which is an alarming signal for clinicians as end-stage liver disease leads to an increased risk of liver damage [87]. Most studies on severity and surveillance and the incidence of liver injury in COVID-19 patients are insufficient; thus, large cohort studies are required to understand the detailed relationship between COVID-19 severity and liver disease and the long-term effect of COVID-19 on liver patients [88].

Another clinical challenge is the lack of therapeutic effects of glucocorticoids in COVID-19 patients with the progression of AIH [89]. Possible reactivation of the hepatitis B virus with some biological drugs, such as tocilizumab and baricitinib, may also lead to liver damage in severe cases. Some drugs, such as tocilizumab and baricitinib, which increase the severity of liver disease, were used for HBV treatment and were also used for targeting viral replication. However, the exact mechanism of action of these drugs in combination for treating chronic liver disease remains unclear and is a major challenge for combination therapy [89]. Moreover, it is also unknown whether a SARS-CoV-2 infection enhances cholestasis in patients with underlying cholestatic hepatic disease.

Liver transplant patients with COVID-19 have a high risk of severe complications and mortality as these patients receive immunosuppressive drugs that affect hepatic function [90]. Increasing the dosage of immunosuppressive drugs can cause adverse reactions that remain challenging in immunotherapy. For example, tacrolimus is an immunosuppressive drug that increases the risks of kidney failure and hypertension. Alemtuzumab promotes the development of ulcer cancer and leukopenia [91,92]. Therefore, novel therapeutics should be designed for liver transplant recipients emphasising potential drug side effects.

## 6. Conclusions and Future Perspectives

The increased number of cases of liver injury in COVID-19 has become a global problem, especially in patients with compromised immune systems. Liver injury in COVID-19 patients is induced through multiple factors, including a direct attack of SARS-CoV-2 on liver hepatocytes, hypoxia reperfusion dysfunction, cytokine release syndrome, drug-induced liver injury caused by lopinavir and ritonavir, immune-mediated inflammation, and coagulopathy. COVID-19 patients with preexisting liver injury exhibit clinical manifestations, including elevation in ALT, AST, GGT, bilirubin, and failure in hepatic function. These patients have a high risk of developing NAFLD, HCC, AIH, and ALD diseases. Patients with SARS-CoV-2 have liver dysfunction, especially when they have chronic liver diseases that influence the disease prognosis. Such patients should be admitted to the hospital, LFTs should be regularly monitored, and they should continue anti-viral drugs and take precautions to prevent the recurrence of chronic liver disease. COVID-19 patients with LT still need to take caution as there are high chances of transmission of viral infections to healthcare workers. COVID-19 patients with CLD should use telemedicine and may continue upon the advice of an expert physician. However, further studies are needed to explore the cellular and molecular events in COVID-19 patients with liver injury. COVID-19 patients with chronic liver disease and liver transplant patients should undergo vaccination in order to avoid the risk of transmission of infection to healthy individuals. Although the mechanism of action of SARS-CoV-2 attack on liver hepatocytes through ACE2 still lacks the necessary information. L-SIGN and CD147, which are also not fully understood, may act as alternative receptors, and further studies are needed to confirm the association between SARS-CoV-19 replication and liver injury.

## Figures and Tables

**Table 1:** The reported studies indicate drug-induced liver injury in COVID-19 patients.

Study Type	Enrolled Patients	Medication Type	Dose Concentration (mg)	Duration	Drug-Induced Liver Injury	Outcomes	References
Case Study	4	Remdesivir, Hydroxychloroquine	10	Daily	Elevated AST and ALT (5–8 times)	Liver cirrhosis, cardiac failure, and organ dysfunction	[44]
Randomized design	158	Lopinavir, Remdesivir, Corticosteroids	100	Daily	AST/ALT (5 times folds)	Adverse effects (Liver dysfunction and circulatory failure) were observed in 102 patients	[45]
Case Study	One patient susceptible to medication	Chloroquine, Methylprednisolone, Tocilizumab	500	Daily	Transaminase elevated (10 times folds)	Autoimmune liver disease, cytokine release syndrome	[42]
Cross-sectional	417	Ribavirin, Ritonavir	Undetected thought the study	Throughout treatment	AST/ALT (3 times folds)	Hepatocellular carcinoma, NAFLD	[4]
Retrospective	179	Tocilizumab	800	24 h	Transaminase sharply after dosage	Liver cirrhosis	[46]
Case Study	One patient susceptible to study	Favipiravir	6000	Daily	Transaminase was significantly elevated	Cholestasis liver disease	[47]
Case Study	5	Remdesivir	200	Daily	ALT was significantly elevated	Advanced liver disease and renal failure	[48]

## Data Availability

Not applicable.
